# Evidence-Based Analysis of the Critical Steps of Radical Cystectomy for Bladder Cancer

**DOI:** 10.3390/jcm12216845

**Published:** 2023-10-30

**Authors:** Vincent D. D’Andrea, Kevin Melnick, Kendrick Yim, John Ernandez, Nnamdi Onochie, Timothy N. Clinton, Graeme S. Steele, Mark A. Preston, Adam S. Kibel, Matthew Mossanen

**Affiliations:** 1 Division of Urology, Department of Surgery, Harvard Medical School, Brigham & Women’s Hospital, Boston, MA 02115, USA; vdandrea@bwh.harvard.edu (V.D.D.);; 2 Dana-Farber Cancer Institute, Harvard Medical School, Boston, MA 02115, USA

**Keywords:** radical cystectomy, urologic oncology, bladder cancer, surgical technique, surgical outcomes

## Abstract

Radical cystectomy (RC) is an integral part of the management of patients with advanced-stage bladder cancer. This major oncologic operation is prone to complications resulting in morbidity and mortality. We analyzed the critical steps of open RC, performed an evidence-based review of these steps, and discussed our experience and approach. We conducted a literature review of the open RC technique, identified the critical steps that consistently appeared across different sources, and organized these steps into a framework. PubMed was queried with the critical steps as keywords for relevant articles published from 1 January 2013 to 1 August 2023. We utilized this query to conduct a systematic review of the literature using the outcomes of overall survival and 90-day complication rate. We developed the “Summary for the 10 Critical Operative Steps of Radical Cystectomy”, a concise guide to the approach to open RC. When available, an evidence-based analysis of each critical step was performed. We also included additional components of cystectomy optimization such as pre-habilitation in the preoperative phase, standard versus extended lymphadenectomy, the vaginal-sparing approach to female radical cystectomy, patient-reported outcomes following urinary diversion, the use of a mesh for stoma formation, and the use of the ERAS protocol for postoperative care. An evidence-based assessment of RC may help provide valuable information to optimize surgical techniques and patient outcomes.

## 1. Introduction

In 2018, bladder cancer (BC) was the tenth most common malignancy worldwide [1]. In the United States (US) in 2023, there were an estimated 82,290 new cases and 16,710 estimated deaths due to bladder cancer [2]. The demographics of BC are evolving, which may be in part due to improvements in our understanding of treatment approaches and the human genome (such as germline and acquired gene mutations which may predispose an individual to BC) and changing worldwide risk factors [3]. For example, roughly half of BC cases are related to cigarette smoking, the rate of which has declined in recent years in many countries [4]. Similarly, workplace regulations have reduced exposure to known high-risk carcinogens associated with BC [5].

Most BC cases are urothelial carcinoma (UC) in subtype (the remainder includes squamous cell, sarcoma, lymphoma, and adenocarcinoma). Most bladder cancers are diagnosed after patients present with hematuria. Cases are confirmed after transurethral resection of bladder tumors (TURBT) and approximately 25% of these are muscle-invasive BC (MIBC) at diagnosis [6]. Radical cystectomy (RC) with pelvic lymph node dissection (PLND) remains the gold standard surgical treatment for MIBC and >10,000 surgeries are performed each year in the US [7]. The procedure has traditionally been performed using an open approach and minimally invasive approaches are becoming increasingly utilized. Regardless of the surgical approach, morbidity from radical cystectomy is consistent and considerable with an estimated 20–30% re-admission rate and 20% of patients requiring further invasive procedures [8]. A significant interest in minimally invasive surgery (MIS) has arisen in the last two decades in an effort to decrease morbidity, and open RC still remains commonly utilized with respect to laparoscopic and robotic techniques.

Several clinical trials to date have compared the open approach and the robotic approach to RC. These trials are summarized in Table 1. These have shown, in short, that there are equivalent oncologic outcomes between the approaches with mild significant improvements for length of stay, complication rate, operative time, and blood loss for robotic approaches.

A resounding theme in the surgical outcomes literature of bladder cancer is a recent pattern towards centralization in the surgical treatment of bladder cancer to large centers with high-volume surgeons and dedicated treatment pathways [16]. Particularly as it pertains to open RC, there has been a focus on the importance of surgical volume and the mastery of techniques to improve morbidity associated with open RC [17]. Herein, we analyze the critical steps of open RC. We review the variation in these critical steps with evidence-based comparisons and discuss our experience and approach. In doing so, we aim to summarize the technical aspects of RC that may be valuable in better understanding the steps and techniques of this complicated operation.

## 2. Materials and Methods

In this review, we conducted a comprehensive analysis of the critical steps involved in open RC. Our primary objective was to provide a succinct summary that can be used as a tool for a better understanding of the surgical technique. We began by conducting an extensive review of the existing recent literature on the open RC technique. From the collected literature, we identified the critical steps that consistently appeared across different sources. These steps were further organized into a step-by-step framework. PubMed was again queried with the critical steps as keywords for relevant articles published in the past ten years dating from 1 January 2013 with a cutoff date of 1 August 2023. We utilized the advanced search terms “(bladder cancer) AND (open radical cystectomy) AND (KEYWORD)”, where KEYWORD was each of the critical steps we identified. We used these keyword searches to build a comprehensive literature library from which we reviewed the identified critical steps in an evidence-based fashion.

We then utilized the search criteria “(bladder cancer) AND (open radical cystectomy) AND (urinary diversion)” to conduct a systematic review of the literature with the primary outcomes of 2-year overall survival (OS), total 90-day complications, and major 90-day complications. 

Variations in techniques and approaches were noted and evidence-based comparisons were made to highlight the differences among surgeons’ practices. To supplement the literature findings, we incorporated the clinical experience and insights of surgeons who commonly perform the operation in our healthcare system. These key points were integrated into the analysis to provide a well-rounded view of the procedure. We further synthesized the literature review into a concise and informative resource that may help improve patient outcomes or serve as a guide for surgeons learning this operation.

## 3. Results

**Figure 1:**
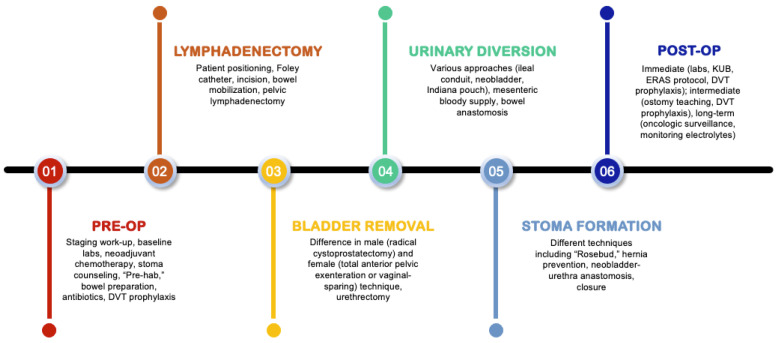
The critical steps of open RC.

Through the literature review described above, the critical steps of open RC were identified as: (1) preoperative considerations, (2) lymphadenectomy, (3) bladder removal, (4) urinary diversion, (5) stoma formation, and (6) postoperative considerations (Figure 1). 

**Figure 2:**
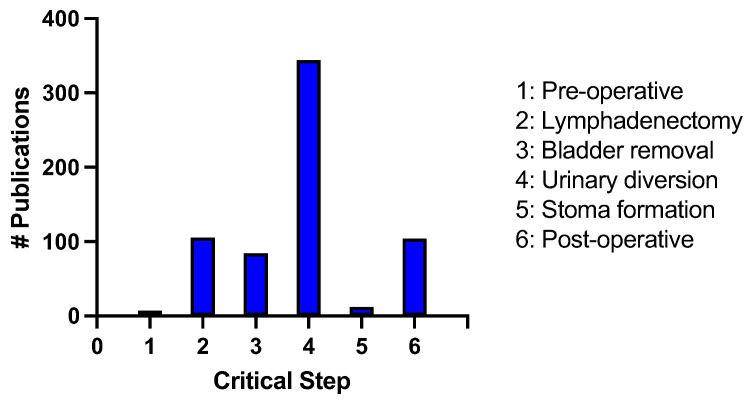
Number of publications identified from 2013–2023 in an advanced PubMed search using the critical steps of radical cystectomy as keywords.

We utilized the critical steps identified to undertake a comprehensive search of the literature between the years of 2013–2023 using the critical steps as keywords. We recorded the number of publications during this 10-year period with the following results: (1) preoperative considerations = 7; (2) lymphadenectomy = 105; (3) bladder removal = 84; (4) urinary diversion = 347; (5) stoma formation = 12; (6) postoperative considerations = 104 (Figure 2).

**Figure 3:**
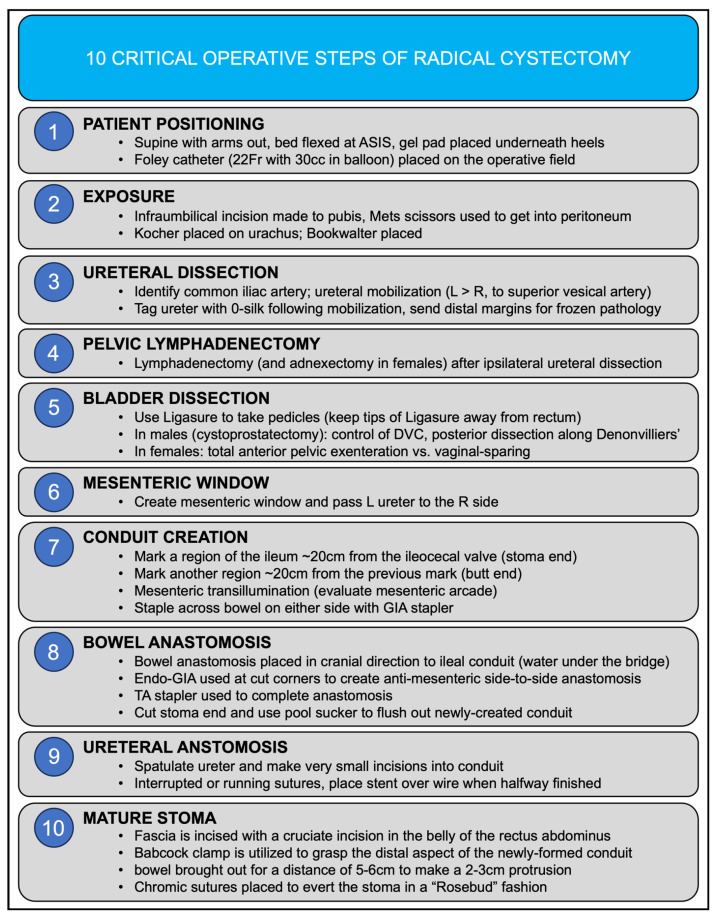
Quick sheet summary for the ten critical operative steps of radical cystectomy.

We subsequently discuss each critical portion of open RC in detail, including an evidence-based comparison of the approaches to various steps of the operation using the publication library formulated above. A key component of this resource was the development of a “Quick Sheet”, a concise single-page guide to the framework and approach to the technique for open RC (Figure 3). This sheet discusses and summarizes the ten most critical operative steps of radical cystectomy.

**Figure 4:**
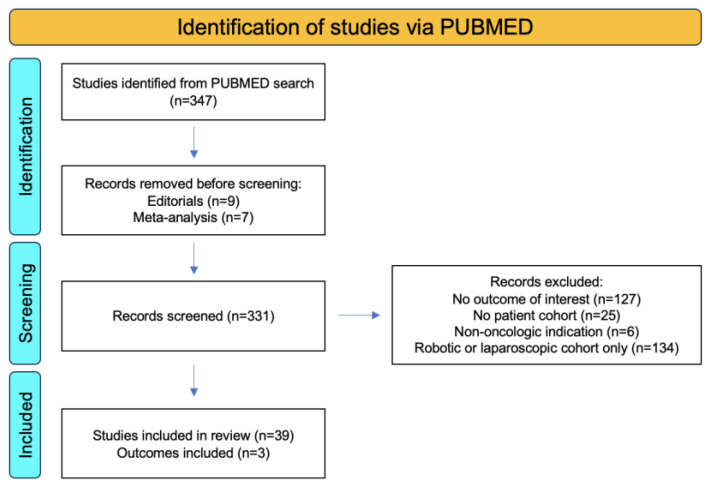
PRISMA flowchart showing identification, screening, and inclusion criteria for systematic reviews of urinary diversion in bladder cancer using the primary outcomes of OS, total 90-day complications, and major 90-day complications.

Using the search criteria “(bladder cancer) AND (open radical cystectomy) AND (urinary diversion)”, we conducted a systematic review of the literature from 2013–2023 with the primary outcomes of overall survival (OS), total 90-day complications, and major 90-day complications. A total of 347 studies were identified using this search criteria. Out of these, 30 were prospective clinic trials. Editorials (n = 8) and meta-analyses (n = 7) were removed from the search and a total of 331 studies were screened. Excluded studies included those that did not include the outcome of interest (n = 127), those with no patient cohort (n = 25), those that involved cystectomies completed for non-oncologic indications (n = 6), and those that included only robotic and/or laparoscopic patient cohorts (n = 134). This resulted in 34 total studies which were included in the systematic review (Figure 4).

**Figure 5:**
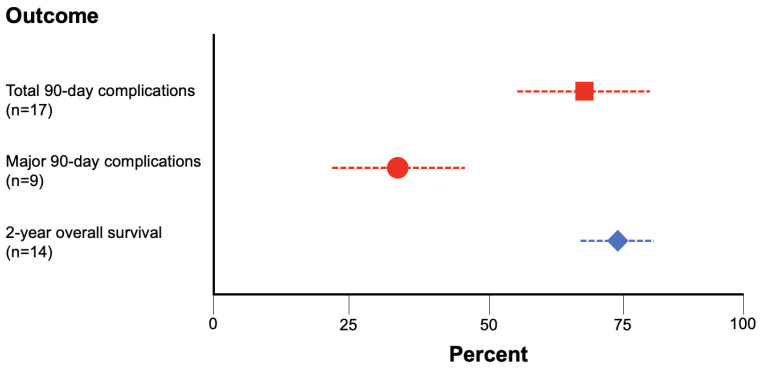
Rates of 2-year OS (blue diamond), total 90-day complications (red square), and major 90-day complications (red circle) for RC gathered via systematic review of the literature.

Using these criteria, we found that the 2-year OS for RC was 73.3% ± 8.1% (n = 14 studies, 2870 total patients), the total 90-day complication rate for RC was 70.6% ± 14.4% (n = 17 studies, 3246 total patients), and the major 90-day complication rate for RC was 32.7% ± 15.8% (n = 9 studies, 1699 total patients) (Figure 5).

### 3.1. Preoperative Considerations

Patients undergoing RC should undergo a complete metastatic and staging evaluation and imaging with computed tomography (CT) and/or magnetic resonance imaging (MRI). Particular attention should be paid to lymphadenopathy, local extension of tumors, and anatomic abnormalities. Neoadjuvant chemotherapy should be considered when indicated. A bowel preparation is not necessary for small intestinal conduits and our institutional practice is to omit the bowel prep unless there are extenuating circumstances (e.g., possible rectal invasion or planned total exenteration). All patients should be marked preoperatively for the potential urostomy site.

Many surgical centers have a preoperative teaching session for post-cystectomy care and stoma management and this practice has been well-supported in the literature. For example, Smelser et al. (2023) implemented a comprehensive pre-rehabilitation program with a pre-defined order set for all patients undergoing RC between February to December 2021 which showed the feasibility and a modest improvement in resource consumption and complication rate [18]. Minnella et al. (2021) implemented a prospective clinical trial that included multimodal pre-rehabilitation, including aerobic and resistance exercise, diet therapy, and relaxation techniques which resulted in a faster functional recovery after RC [19]. Our institutional practice is to implement a multi-disciplinary preoperative course covering lifestyle changes that patients can make to help improve their health prior to surgery and recovery.

Broad-spectrum antibiotics covering gram-negative, gram-positive, and anaerobic organisms are administered 60 min before skin incision. This can generally be achieved with ceftriaxone and metronidazole. Standard deep vein thrombosis (DVT) prophylaxis with sequential compression devices (SCDs) and heparin should be utilized per institutional practice.

### 3.2. Pelvic Lymphadenectomy

In general, patients are positioned with the bed flexed and a lower midline incision is made. The bowel is mobilized to identify the ureters; the distal ends of the ureters are ligated and marked bilaterally, and the far distal margin is sent for frozen pathology. A pelvic lymphadenectomy is performed bilaterally. The anatomic boundaries of the standard pelvic lymphadenectomy consist of the genitofemoral nerves laterally, the internal iliac artery medially, Cooper’s ligament inferiorly, and the point at which the ureter crosses the common iliac artery superiorly [20]. Care should be taken to avoid injury to the obturator nerve. 

Surgical quality as measured by nodal yield has demonstrated a survival benefit in bladder cancer and Herr et al. (2004) showed that patients with a lymph node yield >10 had improved 5-year survival [21]. In cases of advanced disease, an extended dissection inclusive of the entire common iliac lymph node packet and the presacral lymph node packet can be obtained. Wang et al. (2019) conducted a meta-analysis of 10 studies that showed that extended lymphadenectomy was correlated to higher recurrence-free survival and disease-specific survival in patients with a similar postoperative complication profile to standard lymphadenectomy [22]. In contrast, Lerner et al. (2023) recently reported the SWOG S1011 trial which showed no improvement in disease-free survival and overall survival in patients undergoing extended versus standard pelvis lymphadenectomy, and that extended lymphadenectomy was associated with greater morbidity and peri-operative mortality [23]. Similarly, Gschwend et al. (2019) showed in a prospective clinical trial that extended lymphadenectomy during RC failed to improve recurrence-free survival (RFS), cancer-specific survival (CSS), or overall survival (OS) [24].

### 3.3. Bladder Removal

Following pelvic lymphadenectomy, the procedure for bladder removal diverges for males and females. In males, the anterior vascular pedicle blood supply is ligated and the posterior dissection along the rectum is completed and carried to the level of the prostate with the incision of Denonvilliers fascia. The anterior dissection of the prostate is then carried out in a fashion similar to a radical prostatectomy with an incision of the endopelvic fascia and ligation of the dorsal venous complex. Care must be taken to avoid injury to the rectum at this point, particularly if thermal sealing instruments are utilized in the dissection. If neobladder formation is planned, adequate urethral length must be maintained and a frozen section must be sent for pathology. Importantly, urethrectomy may be indicated in males with overt evidence of urethral involvement or prostatic involvement of disease. 

In females, RC has historically included a total anterior pelvic exenteration of the bladder, urethra, anterior vagina, uterus, and cervix. Increasing effort has been undertaken to study a vaginal-sparing approach to RC in females who wish to maintain sexual function. This portion of the operation varies from the standard approach in that the space between the bladder neck and vagina is operated on without injuring the vaginal vault [25]. Retrospective studies have been performed showing that vaginal-sparing RC has equivalent oncologic outcomes and improved sexual function, quality of life, and psychological outcomes. For example, Patel et al. (2022) showed that the vaginal-sparing approach did not increase positive margin rates or decrease RFS, CSS, or OS in a single-institution retrospective study [26]. There is still a need for prospective clinical trials in this area.

### 3.4. Urinary Diversion

The most frequently used segment of the bowel for urinary diversion is the ileum. The colon, stomach, and rectum can also be used in diversion techniques, although this is less common. This review will focus on small bowel segments for urinary diversion. No matter which intestinal segment is utilized, there are many principles that guide the successful completion of a urinary diversion. Adequate exposure is necessary to avoid strangulation of the bowel and perform the anastomosis. Second, a good blood supply is critical to ensure proper recovery of the bowel postoperatively. General principles of vascular anatomy suggest that 15 cm of small bowel can survive laterally to a straight vessel which arises from the anastomoses of mesenteric arcades. However, it is thought that no more than 8 cm of mesentery should be cleared from the end of a small bowel segment to allow for adequate perfusion and minimize the risk of necrosis. Third, care should be taken to avoid the local spillage of enteric contents during the anastomosis. Fourth, adequate apposition of serosa from both sides of the anastomosis should be ensured and care should be taken to not tie the anastomotic sutures so tightly that the tissue is strangulated. Finally, the realignment of the two opposing mesentery segments should be parallel to avoid twisting [27]. We share our step-by-step technique for ileal conduit formation below.

First, a mesenteric window is fashioned and the left ureter is passed to the right side. The cecum and terminal ileum are identified and a point roughly 20 cm from the terminal ileum is identified and marked. Another point 20 cm proximal from this point is then marked. The space between the markers is the portion of the bowel which will be formed into the conduit. The conduit is held up to the light to assess the mesenteric arcades and a stapler is utilized on both sides to incise this portion of the bowel with adequate blood supply. The bowel anastomosis is then fashioned over the ileal conduit which will have the ureters anastomosed in the next step (using the mantra “water under the bridge”). This is completed with an endo-GIA stapler to form a side-to-side anastomosis on the anti-mesenteric side of the conduit. The uretero-intestinal anastomosis is then fashioned bilaterally. If an ileal conduit is being performed, the next step of the operation is stoma formation (discussed in the following section). If an ileal neobladder is being performed, the surgeon then proceeds to the urethral-intestinal anastomosis.

**Figure 6:**
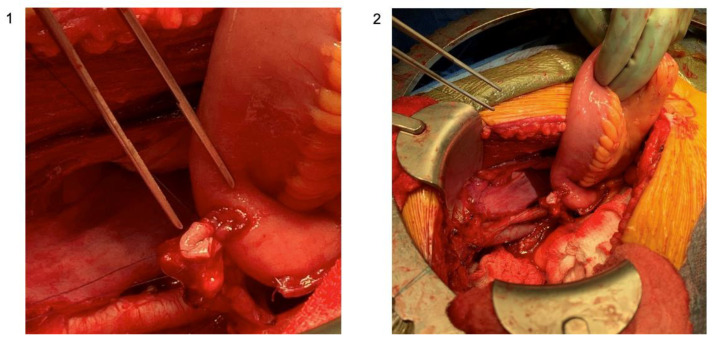
Set-up of the uretero-intestinal anastomosis with ureteral and bowel mucosal apposition; (**1**) zoomed-in photo with attention to the suture placement; (**2**) zoomed-out photo with attention to the surgical set-up (original images; courtesy: Dr. Matthew Mossanen).

The uretero-intestinal anastomosis is one of the most technically complex and demanding portions of the procedure. The ureters are spatulated anteriorly and sharp scissors are used to make a small incision into the conduit. Our practice is to tie down into the ureter to the conduit with a 4-0 Maxon or Vicryl suture with eight knots in an interrupted fashion. When half of the sutures are placed, a wire is passed up to the renal pelvis through the conduit with a plastic suction and stents are placed up over the wire. The anastomosis is then completed. The key components of uretero-enteric anastomoses are summarized by the acronym SMART and include spatulation, mucosal apposition, rotational (avoiding twisting of the ureter), and tension-free. The set-up and completed utero-intestinal anastomosis is shown in Figure 6.

Given the extensive changes to the urinary and genital anatomy following radical cystectomy, healthcare-related quality of life has been an important marker for the overall “success” of the operation and many studies have probed this phenomenon. For example, Singh et al. (2014) published a prospective study that showed that a neobladder is better than an ileal conduit in terms of physical functioning, social functioning, and financial expenditure [28]. More recently, Clements et al. (2022) published a prospective cohort trial showing that patients undergoing ileal conduit and neobladder surgery do not experience large decreases in health-related quality of life following surgery, with most areas assessed as returning to or exceeding the baseline, except for sexual function and body image [29]. Our practice is to counsel patients extensively on the pros and cons of each technique, particularly if they are candidates for either, and come to a decision on the surgical approach by utilizing a process of shared decision-making.

### 3.5. Stoma Formation

Stoma formation is necessary for techniques associated with both an ileal conduit and an Indiana pouch. All stomas should be placed through the belly of the rectus muscle and be located at the peak of the infraumbilical fat roll. The fascia is incised with a cruciate incision with the rectus muscle split. To fashion the stoma, the peritoneal cavity is entered, and a Babcock clamp is utilized to grasp the distal aspect of the newly formed conduit. Two types of abdominal stomas are possible: a Turnbull (loop) stoma or a protuberant stoma. When possible, protruding (rosebud) stomas are preferred when a collection device is worn (such as with an ileal conduit).

To create a rosebud stoma, the bowel is brought out through the cruciate incision for a variable distance (usually a few centimeters) to allow for stoma protrusion. Two 3-0 Vicryl sutures are placed after the stoma is brought through and everted. It is important to verify the proper alignment of the mesentery prior to placing these sutures. Four additional sutures are placed in quadrants through the full thickness of the bowel edge and the subcuticular layer of the skin. When these sutures are tied, the bowel is everted to form a rosebud.

Parastomal hernia following ileal conduit is a known complication that may impair postoperative quality of life. Dewulf et al. (2022) published a systematic review of the use of mesh in preventing parastomal hernia formation. Among other findings, five studies reported on the use of keyhole mesh in a retro-muscular position for the prevention of parastomal hernia with favorable results in the mesh group without an increase in mesh-related complications [30]. Similarly, Leidberg et al. (2020) showed in a prospective randomized clinical trial that the prophylactic use of mesh during ileal conduit formation decreases the risk of parastomal hernia without increasing complications related to the mesh [31]. It is not our standard practice to leave the prophylactic mesh following ileal conduit surgery.

### 3.6. Postoperative Considerations

Our institutional practice is to leave a drain at the site of the ureteral-intestinal anastomosis following ileal conduit creation. In the immediate postoperative period, we obtain labs to assess for leukocytosis, bleeding, and kidney/electrolyte imbalance and KUB to assess for stent positioning. We institute a customized diet advancement protocol for each patient and generally have a low threshold for placing a nasogastric tube postoperatively if there is a concern for the ileus.

Much of the morbidity and mortality of radical cystectomy involves the bowel, and 75% of lethal complications that occurred in the postoperative period were related to the bowel. Tinoco et al. (2021) summarize the complications associated with urinary diversions following radical cystectomy [32]. Early complications include ileus, urinary tract infection, and urine leakage. Importantly, there is evidence that alvimopan (a peripherally acting µ-opioid receptor antagonist that prevents the binding of opioids in the intestinal tract) leads to decreased ileus formation following radical cystectomy [33]. There is evidence to suggest that ERAS (enhanced recovery after surgery) protocols are beneficial following radical cystectomy with urinary diversion; however, most of the data is extrapolated from the literature pertaining to colorectal surgery [34]. We discharge our patients following the complete return of bowel function and following an intensive postoperative ostomy teaching course. It is also our standard practice to discharge patients with a single month of postoperative anticoagulation for deep vein thrombosis (DVT) prophylaxis.

Late complications include ureteral stricture, bowel obstruction, renal failure, recurrent urinary tract infection, parastomal hernia, and nutritional deficiencies. Loss of portions of the ileum results in long-term nutritional problems because of lack of vitamin B12 absorption, diarrhea because of lack of bile salt reabsorption, and fat malabsorption. Postoperative adhesive bowel obstruction occurs in roughly 12% of patients following radical cystectomy within 5 years [35].

## 4. Discussion

This study provides a comprehensive analysis of the essential procedural elements involved in open RC. The primary objective of this study was to establish a concise evidence-based resource for surgeons learning and optimizing their surgical techniques in this domain. For those in training or studying this surgery, the “Summary for the 10 Critical Operative Steps of Radical Cystectomy” depicted in Figure 2 may be useful. This succinct one-page guide encapsulates the key principles and methodological aspects of RC for open surgery as an additional reference.

The critical steps of open RC were identified as: (1) preoperative considerations, (2) lymphadenectomy, (3) bladder removal, (4) urinary diversion, (5) stoma formation, and (6) postoperative considerations. We focused our attention on various evidence-based components of these critical steps, including pre-rehabilitation in the preoperative phase, standard versus extended lymphadenectomy, the vaginal-sparing approach to female radical cystectomy, patient-reported outcomes following urinary diversion, the use of mesh for stoma formation, and the use of the ERAS protocol for postoperative treatment.

We also conducted a systematic review of the literature to determine the key outcome measures of radical cystectomy in the past 10 years in an evidence-based manner. While the total 90-day complication rate is high, the major complication rate at 90 days is roughly half, indicating that many of the complications that occur in the first 3 months of surgery are relatively minor. This finding underscores the safety and reliability of radical cystectomy in the treatment of bladder cancer, while also emphasizing the necessity for training and continued assessment amongst surgeons who perform the operation. The 2-year overall survival serves as an interesting benchmark in patients who undergo radical cystectomy, and further studies are needed to see if this rate may be influenced by other factors such as stage at diagnosis or prior radiation therapy.

Important limitations of our study include the nature of our search criteria and our search timeline for the literature review, which may have limited the characteristics and the quantity of our results. Furthermore, we acknowledge that our institutional observations may have limitations that do not apply to a general scope and that surgeon expertise and surgical center may impact the applicability of the presented work. Each surgeon and each patient are unique and no one technique or approach is best. The reality is that surgeon volume and experience remain the most critical elements of cystectomy. Nonetheless, we hope that this study opens avenues for further research into the various critical steps of open RC and serves as a useful guide or tool for those learning or performing this complex operation.

## 5. Conclusions

RC remains an essential component in the management of bladder cancer. Regardless of recent innovations or approaches, the operation is still a major undertaking with the possibility of complications causing morbidity and mortality. Given its complexity, surgeon expertise and mastery of technique have become paramount in improving outcomes regardless of surgical approach. 

Our goal in this evidence-based analysis of the critical steps of open RC is to form a practical and succinct tool for surgeons to enhance their practice in the treatment of patients with bladder cancer. We further sought to identify the baseline overall survival and complication rate via a systematic review of the literature. Through this study, we identified six critical steps in the open RC procedure: preoperative considerations, lymphadenectomy, bladder removal, urinary diversion, stoma formation, and postoperative considerations. Each of these steps has been dissected and the “Summary Sheet” condenses these steps into a concise document, which can improve the surgical preparation of surgeons performing and learning to perform this operation.

## Figures and Tables

**Table 1:** Prospective clinical trials comparing robotic RC to open RC.

Study	Year	Total Patients	Primary Outcome(s)	Observation between Robotic and Open RC
Nix et al. [9]	2010	41	Lymph node yield	No difference
Bochner et al. [10]	2015	118	90-day complication rate	No difference
CORAL [11]	2016	164	30- and 90-day complication rate	Favored robotic vs. open for 30-day complication rate No difference for 90-day complication rate
Bochner et al. (update) [12]	2018	118	Recurrence; cancer-specific survival; overall survival	No difference
RAZOR [13]	2019	350	Two-year progression-free survival	No difference
CORAL (update) [14]	2020	60	Recurrence; cancer-specific survival; overall survival	No difference
iROC (intracorporeal diversion) [15]	2022	317	Median number of days alive and outside of the hospital within 90 days of surgery	Favored robotic (82 days) vs. open (80 days) approach

## Data Availability

No new data were created or analyzed in this study. Data sharing is not applicable to this article.
